# Association between genetic variation of complement *C3* and the susceptibility to advanced age-related macular degeneration: a meta-analysis

**DOI:** 10.1186/s12886-018-0945-5

**Published:** 2018-10-23

**Authors:** Jun Zhang, Shuang Li, Shuqiong Hu, Jiguo Yu, Yi Xiang

**Affiliations:** 1 Department of Ophthalmology, the Central Hospital of Wuhan, Tongji Medical College, Huazhong University of Science and Technology, NO, 26 Shengli Street, Wuhan, 430014 Hubei Province China; 2 Department of Ophthalmology, the Jingzhou aier eye hospital, Jingzhou, Hubei Province China

**Keywords:** Age-related macular degeneration, *C3* gene, Polymorphism, Meta-analysis

## Abstract

**Background:**

The purpose of this study is to discuss whether genetic variants (rs2230199, rs1047286, rs2230205, and rs2250656) in the *C3* gene account for a significant risk of advanced AMD.

**Methods:**

We performed a meta-analysis using electronic databases to search relevant articles. A total of 40 case-control studies from 38 available articles (20,673 cases and 20,025 controls) were included in our study.

**Results:**

In our meta-analysis, the pooled results showed that the carriage of G allele for rs2230199 and the T allele for rs1047286 had a tendency to the risk of advanced AMD (OR = 1.49, 95% CI = 1.39–1.59, *P* < 0.001; OR = 1.45, 95% CI = 1.37–1.54, P < 0.001). Moreover, in the subgroup analysis based on ethnicity, rs2230199 and rs1047286 polymorphisms were more likely to be a predictor of response for Caucasian region (OR = 1.48, 95% CI = 1.38–1.59, *P* < 0.001; OR = 1.45, 95% CI = 1.37–1.54, P < 0.001). Besides, pooled results suggested that the G allele of rs2230199 could confer susceptibility to advanced AMD in Middle East (OR = 1.62, 95% CI = 1.33–1.97, *P* < 0.001).

**Conclusion:**

In our meta-analysis, *C3* genetic polymorphisms unveiled a positive effect on the risk of advanced AMD, especially in Caucasians. Furthermore, numerous well-designed studies with large sample-size are required to validate this conclusion.

**Electronic supplementary material:**

The online version of this article (10.1186/s12886-018-0945-5) contains supplementary material, which is available to authorized users.

## Background

Age-related macular degeneration (AMD) is a complex and progressive retinal disorder influenced by family history, aging, race, smoking and diet, which caused irreversible visual impairment in a growing number of elderly persons [1, 2]. The early stage of AMD is characterized by pigmentary abnormalities of the retinal pigment epithelium (RPE) and extracellular deposits called drusen under the retina [3]. As the condition progresses, two advanced forms of this disease are developed: extensive pigment epithelium atrophy (geographic atrophy or dry AMD) or subretinal choroidal neovascular membrane (exudative or wet AMD). Although constituting only 10–15% of all AMD cases, advanced forms account for nearly 80% of AMD-related blindness in western countries [4]. It has been reported that the prevalence of advanced AMD is estimated at 3% in people aged > 65 years old, rising to 11% in those > 85 years old in developed world [5]. While, the pooled prevalence of advanced AMD is 0.56% among aged 40–79 years in Asian countries [6].

Advanced AMD has been implicated with important risk factors listed above, it is a multifactorial disease which influenced by a combination of environmental and genetic susceptibility [1, 3, 7, 8]. Although the well-defined pathogeny of advanced AMD remains to be unresolved, genetic association studies have provided consequential insights into the molecular basis of advanced AMD. Several genes at chromosomal loci 1q32 and 10q26, involving in inflammation and complement activation pathway, have been plausible candidate, as supported by the laboratory research in vitro and vivo that inflammation and immune response related proteins were found in drusen [9–11]. So far, the strongest genetic association has been identified on 1q32 with single nucleotide polymorphisms (SNPs) in complement factor H (*CFH*) gene by candidate region and whole genome association analyses [12, 13].

Apart from CFH, the central element of the complement cascade, complement component C3a has been interconnected with the vascular endothelial growth factor expression, geographic atrophy, retinal pigment epithelium deterioration, and progression to choroidal neovascularization [11, 14, 15]. These studies strongly indicated that aberrant regulation or activation of the complement pathway confer susceptibility to the main mechanism of advanced AMD. As the main regulator of the alternative complement pathway, several genetic variants in *C3* gene have been investigated with advanced AMD in different ethnic groups, the pooled results are incompatible and ambiguous. According to the International HapMap Project database, the human *C*3 gene is located on chromosome 19 and exhibits nine common genetic SNPs (rs2230199, rs1047286, rs2241394, rs2250656, rs344542, rs2230205, rs339392, rs3745565, and rs11569536). Used for screening the electronic database and manual searching, the most widely condidate polymorphisms of the *C3* gene which at least has been surveyed in three pertinent studies are rs2230199, rs1047286, rs2230205 and rs2250656. In order to better understand the genetic risk of *C3* gene in the relationship with exudative AMD, we performed a meta-analysis to illuminate this association and determine whether the genetic variants of *C3* gene conferred susceptibility to advanced AMD.

## Materials and methods

### Literature search

A systematic search of electronic database such as PubMed, Embase, CNKI, Cochrane library and Web of Science was conducted with the following keywords: (“AMD” or “maculopathy” or “macular degeneration” or “age-related maculopathy” or “age-related macular degeneration”) and (“complement 3” or “complement *C3*” or “*C3*” or “complement component 3”) and (“variant” or “mutation” or “genetic” or “SNP” or “polymorphism” or “genetic polymorphism” or “genetic variant” or “single nucleotide polymorphism”). Each database was thoroughly scanned and was up to date as of September 1 2018. Our meta-analysis was mainly focused on case-control studies, without any language limitation imposed in the literature searching.

### Study selection

Retrieved articles were considered eligible for our meta-analysis when they met the following inclusion criteria: (1) investigating the disease risk of *C3* polymorphism with advanced AMD; (2) detailed genetyping data for each site could be acquired to estimate the odds ratio (OR) and 95% confidence interval (CI) based on genetic model contrast; (3) individual for all selected samples met the modified version of the age-related eye disease study (AREDS) grading system as described elsewhere. Major exclusion criteria were limited to several items as follow: (1) overlapping subjects in several articles for the same research group; (2) only focused on families^’^ individuals rather than sporadic advanced AMD patients; (3) abstract from conferences, letters, review articles and case reports. When several articles included some of the same samples, the one with largest individuals and thorough genotype information would be winnowed for our meta-analysis.

### Data extraction

Data from the retrieved studies were extracted independently by two reviewers (J.Z. and S.L.). The following items obtained from each eligible articles included: the first author, the year of publication, country and ethnicity of subjects, information on study design, sample size, genotyping methods and distribution in case and control groups. Two authors carefully inspected the raw statistics and reached a consensus in all aspects. If any disagreement still existed, the third author (S.H.) would be invited to chew over current controversy and resolve the dispute.

### Quality assessment

Quality assessment of the screened studies was also independently conducted by two reviewers (J.Z. and S.L.) in the basis of the HuGENet Handbook [16]. A total of six bias assessment items were refined to investigate the relationship between genes and diseases from this handbook, including bias in selection of cases, bias in selection of controls, bias in genotyping cases, bias in genotyping controls, bias in population stratification, confounding bias, multiple tests, and selective outcome reports. The quality evaluation of every items for extracted articles was defined as “Yes” or“No”. Separately, “Unclear” was designated if there was not enough information to make a decision. A series of corrections and judgements were performed independently by another coauthor (S.H.) if debate still lasted in the assessment. Consensus referring all items was achieved after discussion.

### Statistical analysis

Allele and genotype frequency of each *C3* polymorphic site were counted between cases and healthy controls. The genetic strength association including pooled ORs and 95% CIs was assessed using different genetic models, including allele model (A vs. a), homozygote model (AA vs. aa), heterozygote model (Aa vs. aa), dominant (AA+Aa vs. aa), recessive (AA vs. Aa+aa). The heterogeneity assumption between studies was estimated and evaluated by Cochran’s Q statistic as well as the I^2^ statistic. The result that our *P* value of Q statistic was less than 0.05 or the I^2^ value was greater than 50% suggested apparent heterogeneity, thus a random-effect model was utilized in our model analysis. Otherwise, the fixed-effect model was performed [17]. Sensitivity analyses was conducted to assess the effect of each study and the stability of the pooled ORs by removing included study in turn from the compiled list. Begg’s funnel plots [18] and Egger’s regression test [19] were furthered to detect the potential publication bias. All statistical analysis using two-sided *P* values was executed by STATA 12.0 software (StataCorp LP, College Station, Texas, USA). A significant difference was estimated under the level of 0.05. The final results needed to be tested and verified by two authors (J.Z. and S.L.) respectively.

## Results

### Overall characteristics of selected studies and quality assessment

**Fig. 1:**
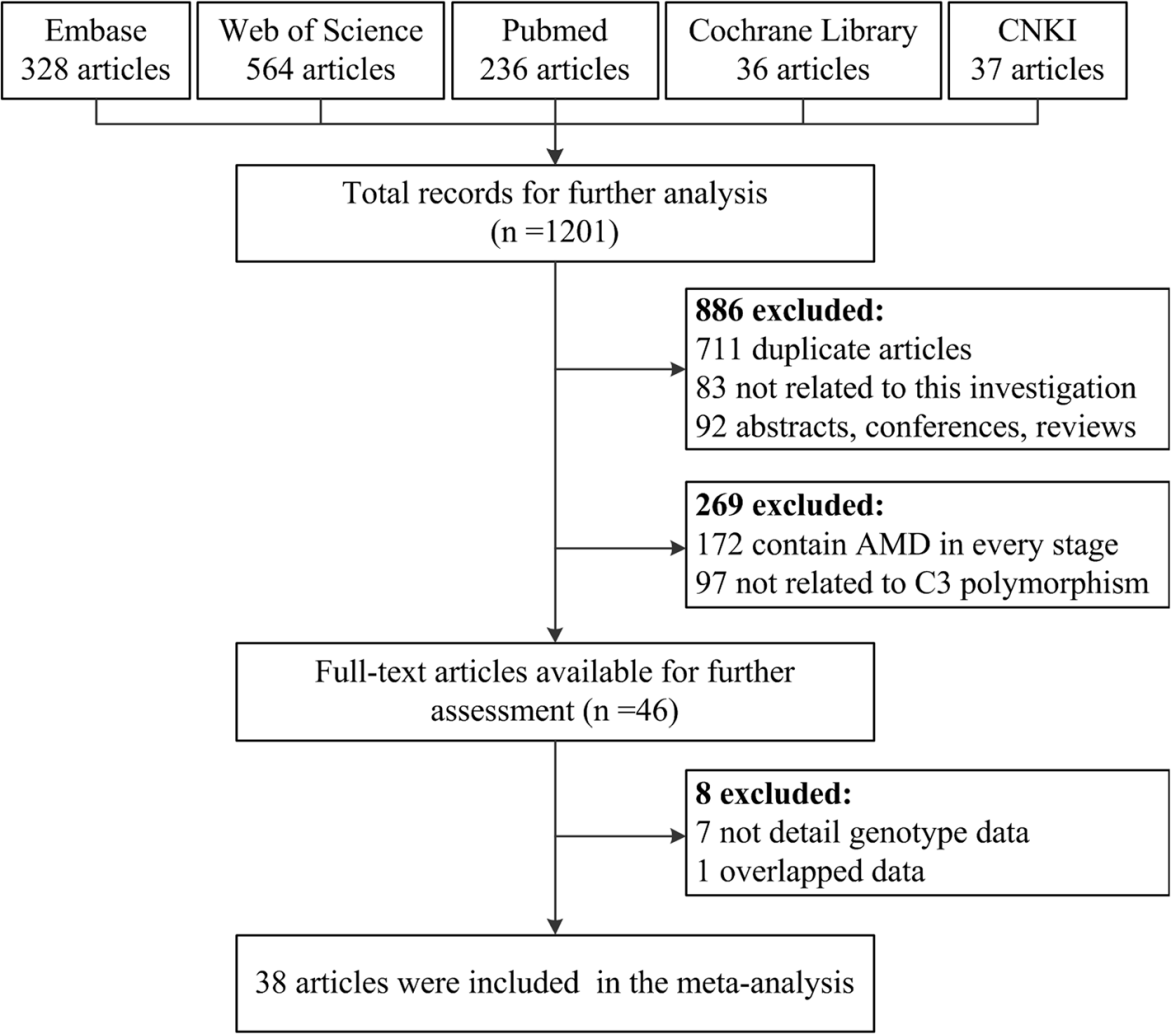
Flow diagram presenting the result of literature searching process in meta-analysis

The flow diagram for literature searching is summarized in Fig. 1. A total of 1201 articles from the five databases (Additional file 1) were filtered by our search method. Of which, 886 studies were excluded for the three aspects: (1) 711 duplicated articles; (2) 83 articles not related to the theme of this investigation; (3) 92 articles mainly referred to abstract, conference, review, and case report. Through our rigorous inspection, 269 ones in the rest of articles were stroke out. Of them, 172 articles were focused on the every stage of AMD but not advanced AMD, 97 articles were not concerned with the association between advanced AMD and *C3* genetic polymorphism. The other 46 full-text articles were left in our meta-analysis. Seven of them did not have detailed genotype data after cautiously reading the included literatures.Besides, two papers were investigated by the same author and the same batch of patients from Iran [20, 21]. We decided to choose the one which had larger samples size and more comprehensive directions. Finally, 40 case-control studies regarding the association of *C3* gene with advanced AMD from 38 available publications were generally contained in our current meta-analysis [4, 20, 22–57]. The common characteristics of each article are generally showed in Table 1. As listed in the table, 30 studies from Caucasian region, 7 studies from East Asian group and 3 studies from Middle East have been chosen in our meta-analysis. The genotyping methods for our whole sample are distinct and the results could be validated in different ways.

**Table 1:** The General Characteristics of All Studies Included in our Meta-Analysis

Refs	Year	Country	Ethnicity	Case/Control	Mean age of AMD	Mean age of control	Typing teaching	Study design
Yate et al. [54]	2007	UK	Caucasian	603/350	79.4 ± 7.2	75.3 ± 7.8	SNaPshot	Sex-,age-,ethnic-,matched
Yate et al. [54]	2007	Scotland	Caucasian	244/351	77.8 ± 9.2	78.0 ± 8.5	TaqMan	Sex-,age-,ethnic-,matched
Maller et al. [41]	2007	America	Caucasian	1238/934	NA	NA	MALDI-TOF MS	Age-,ethnic-, matched
Edwards et al. [31]	2008	America	Caucasian	444/300	NA	NA	Illumina GoldenGate	Age-,ethnic-, matched
Spencer et al. [50]	2008	America	Caucasian	286/701	76.5 ± 7.7	66.9 ± 8.3	TaqMan	Sex-,age-,ethnic-,matched
Scholl et al. [4]	2009	German	Caucasian	99/612	71.8 ± 7.4	76.2 ± 5.3	MALDI-TOF MS	Sex-,age-,ethnic-,matched
Francis et al. [32]	2009	America	Caucasian	211/187	79	74	Sequencing	Age-,ethnic-, matched
Park et al. [44]	2009	America	Caucasian	898/599	80.6 ± 5.0	77.6 ± 4.3	Illumina GoldenGate	Sex-,age-,ethnic-,matched
Despriet et al. [30]	2009	Netherlands	Caucasian	268/173	78.7 ± 7.7	74.1 ± 6.3	TaqMan	Sex-,age-,ethnic-,matched
Bergeron et al. [22]	2009	America	Caucasian	421/215	64.8	66.5	TaqMan	Sex-,age-,ethnic-,matched
Reynolds et al. [47]	2009	America	Caucasian	120/60	82.0 ± 6.9	79.0 ± 4.4	MALDI-TOF MS	Sex-,age-,ethnic-,matched
Pei et al.[45]	2009	China	East Asian	123/130	70.6 ± 8.2	69.2 ± 10.1	MALDI-TOF MS	Sex-,age-,ethnic-, matched
Cui et al. [29]	2010	China	East Asian	150/161	66.6 ± 8.4	65.7 ± 7.8	PCR-RFLP/ Sequencing	Sex-,age-,ethnic-, matched
Zerbib et al. [56]	2010	France	Caucasian	1080/406	79.0 ± 7.4	67.8 ± 7.7	TaqMan	Sex-,age-,ethnic-, matched
McKay et al. [43]	2010	Northern Ireland	Caucasian	437/436	77.6	74.9	SNaPshot	Sex-,age-,ethnic-, matched
Chen et al. [25]	2010	America	Caucasian	2157/1150	78.6	74.1	Illumina GoldenGate	Sex-,age-,ethnic-, matched
Kopplin et al. [37]	2010	America	Caucasian	377/161	NA	NA	Affymetrix GeneChip	Age-,ethnic-, matched
Liu et al. [39]	2010	China	East Asian	158/220	64.0 ± 6.6	63.0 ± 7.8	SNaPshot	Sex-,age-,ethnic-, matched
Yu et al. [55]	2011	America	Caucasian	1082/221	79.5 ± 5.5	77.0 ± 4.6	MALDI-TOF MS	Sex-,age-,ethnic-, matched
Chen et al. [26]	2011	America	Caucasian	1335/509	70.2 ± 5.1	67.0 ± 4.3	SNaPshot	Sex-,age-,ethnic-, matched
Hageman et al. [33]	2011	America	Caucasian	1132/822	76.5 ± 7.1	76.4 ± 7.3	NA	Sex-,age-,ethnic-, matched
Peter et al. [46]	2011	America	Caucasian	48/1260	NA	NA	TaqMan	Age-,ethnic-, matched
Yanagisawa et al. [53]	2011	Japan	East Asian	420/197	74.0 ± 7.5	72.0 ± 6.0	TaqMan	Sex-,age-,ethnic-, matched
Martinez et al. [42]	2012	Spain	Caucasian	259/191	NA	NA	SNaPshot	Age-,ethnic-, matched
Smailhodzic et al. [49]	2012	Netherlands	Caucasian	197/150	NA	NA	Sequencing	Sex-,age-,ethnic-, matched
Buentello et al. [23]	2012	Mexico	Caucasian	159/152	76.4 ± 8.1	73.5 ± 6.8	PCR-RFLP	Sex-,age-,ethnic-, matched
Tian et al. [51]	2012	China	East Asian	535/469	NA	NA	MALDI-TOF MS	Age-,ethnic-, matched
Losonczy et al. [40]	2012	Hungary	Caucasian	275/106	76.0 ± 7.3	79.1 ± 6.1	PCR-RFLP	Sex-,age-,ethnic-, matched
Cipriani et al. [27]	2012	UK	Caucasian	893/2199	78.6 ± 7.5	NA	Illumina BeadChip	Sex-,age-,ethnic-, matched
Jaouni et al. [36]	2012	Israel	Middle East	317/159	78.1 ± 7.6	70.8 ± 8.2	PCR-RFLP	Sex-,age-,ethnic-, matched
Wu et al. [52]	2013	China	East Asian	165/216	69.4 ± 10	64.5 ± 8.0	TaqMan	Sex-,age-,ethnic-, matched
Helgason et al. [35]	2013	Iceland	Caucasian	1107/2869	NA	NA	Illumina BeadChip	Age-,ethnic-, matched
Helgason et al. [35]	2013	America	Caucasian	1525/1288	NA	NA	Illumina BeadChip	Age-,ethnic-, matched
Contreras et al. [28]	2014	Mexico	Caucasian	273/201	76.0 ± 8.0	65.5 ± 9.8	TaqMan	Sex-,age-,ethnic-, matched
Caire et al. [24]	2014	Spain	Caucasian	154/141	75.4 ± 7.2	78.5 ± 7.2	SNaPshot	Sex-,age-,ethnic-, matched
Liu et al. [38]	2014	China	East Asian	200/275	75.3 ± 7.7	74.3 ± 7.6	TaqMan	Sex-,age-,ethnic-, matched
Hautamaki et al. [34]	2015	Finland	Caucasian	301/119	NA	NA	Sequencing	Age-,ethnic-, matched
Saksens et al. [48]	2016	Netherlands	Caucasian	571/900	76.6 ± 8.5	71.3 ± 6.7	KASP	Sex-,age-,ethnic-, matched
Bonyadi et al. [20, 21]	2017	Iran	Middle East	266/228	76.4 ± 7.6	72.7 ± 6.8	PCR-RFLP	Sex-,age-,ethnic-, matched
Habibi et al. [57]	2017	Tunisia	Middle East	145/207	73.1 ± 8.1	NA	PCR-SSP	Age-,ethnic-, matched

### Bias assessment of the included studies

Overall results in Table 2 primarily expound the evaluation of potential sources of bias in our included studies. Overall, the quality of the included studies was consistently absolute. Of the studies, there was no obvious bias in the selection of cases and controls, genotyping controls, population stratification, confounding bias, multiple tests, or selective outcome reports.

**Table 2:** Assessment of potential bias in included studies

Year	First author	Bias in selection of cases	Bias in selection of controls	Bias in genotyping controls	Bias in population stratification	Confounding bias	Multiple test and Selective outcome reports
2007	Yate et al. [54]	NO	NO	NO	NO	NO	NO
2007	Maller et al. [41]	NO	NO	NO	NO	NO	NO
2008	Edwards et al. [31]	NO	NO	NO	NO	NO	NO
2008	Spencer et al. [50]	NO	NO	NO	NO	NO	NO
2009	Scholl et al. [4]	NO	NO	NO	NO	NO	NO
2009	Francis et al. [32]	NO	NO	NO	NO	NO	NO
2009	Park et al. [44]	NO	NO	NO	NO	NO	NO
2009	Despriet et al. [30]	NO	NO	NO	NO	NO	NO
2009	Bergeron et al. [22]	NO	NO	NO	NO	NO	NO
2009	Reynolds et al. [47]	NO	NO	NO	NO	NO	NO
2009	Pei et al. [45]	NO	NO	NO	NO	NO	NO
2010	Cui et al. [29]	NO	NO	NO	NO	NO	NO
2010	Zerbib et al. [56]	NO	NO	NO	NO	NO	NO
2010	McKay et al. [43]	NO	NO	NO	NO	NO	NO
2010	Chen et al. [25]	NO	NO	NO	NO	NO	NO
2010	Kopplin et al. [37]	NO	NO	NO	Unclear	NO	NO
2010	Liu et al. [39]	NO	NO	NO	NO	NO	NO
2011	Yu et al. [55]	NO	NO	NO	NO	NO	NO
2011	Chen et al. [26]	NO	NO	NO	NO	NO	NO
2011	Hageman et al. [33]	NO	NO	NO	Unclear	NO	NO
2011	Peter et al. [46]	Yes	NO	NO	Unclear	NO	NO
2011	Yanagisawa et al. [53]	NO	NO	NO	NO	NO	NO
2012	Martinez et al. [42]	NO	NO	NO	NO	NO	NO
2012	Smailhodzic et al. [49]	NO	NO	NO	NO	NO	NO
2012	Buentello et al. [23]	NO	NO	NO	NO	NO	NO
2012	Tian et al. [51]	NO	NO	NO	NO	NO	NO
2012	Losonczy et al. [40]	NO	NO	NO	NO	NO	NO
2012	Cipriani et al. [27]	NO	Yes	NO	NO	NO	NO
2012	Jaouni et al. [36]	NO	NO	NO	NO	NO	NO
2013	Wu et al. [52]	NO	NO	NO	NO	NO	NO
2013	Helgason et al. [35]	NO	NO	NO	NO	NO	NO
2014	Contreras et al. [28]	NO	NO	NO	NO	NO	NO
2014	Caire et al. [24]	NO	NO	NO	NO	NO	NO
2014	Liu et al. [38]	NO	NO	NO	NO	NO	NO
2015	Hautamaki et al. [34]	NO	NO	NO	NO	NO	NO
2016	Saksens et al. [48]	NO	NO	NO	NO	NO	NO
2017	Bonyadi et al. [20, 21]	NO	NO	NO	NO	NO	NO
2017	Habibi et al. [57]	NO	NO	NO	NO	NO	NO

### Relationship of *C3* gene polymorphisms with advanced AMD susceptibility

Several genetic models for *C3* polymorphisms including rs2230199, rs1047286, rs2230205, rs2250656 were used in our meta-analysis and the combined results are presented in Table 3. Briefly, 36 studies discussed the association of rs2230199 with advanced AMD, 13 studies investigated the relationship between rs1047286 and advanced AMD, 5 studies referred to rs2230205, rs2250656, respectively.

**Table 3:** Main Results of Pooled ORs and Analysis of *C3* gene polymorphism with advanced AMD in our Meta-Analysis

Subgroup	No. of studies	No. of patients		Allele model		Homozygote model		Heterozygote model		Dominant model		Recessive model	
		Cases	Control	OR(95% CI)	P	OR(95% CI)	P	OR(95% CI)	P	OR(95% CI)	P	OR(95% CI)	P
C3 rs2230199 (Associated allele vs. Reference allele: G vs. C)													
Overall	36	34,805	29,499	1.49 (1.39,1.59)	< 0.001	2.33 (1.98,2.74)	< 0.001	1.53 (1.41,1.64)	< 0.001	1.62 (1.51,1.74)	< 0.001	1.99 (1.70,2.34)	< 0.001
Caucasian	28	31,372	26,130	1.48 (1.38,1.59)	< 0.001	2.20 (1.87,2.60)	< 0.001	1.55 (1.43,1.67)	< 0.001	1.63 (1.51,1.75)	< 0.001	1.88 (1.59,2.21)	< 0.001
East Asian	5	2122	2388	1.11 (0.56,2.20)	0.76	–	–	1.32 (0.91,1.93)	0.144	1.49 (1.04,2.15)	0.032	5.60 (1.57,19.9)	–
Middle East	3	1311	981	1.62 (1.33,1.97)	< 0.001	–	–	1.07 (0.66,1.73)	0.798	1.49 (0.95,2.34)	0.085	25.5 (3.35,194)	–
C3 rs1047286 (Associated allele vs. Reference allele: T vs. C)													
Overall	13	16,232	16,222	1.45 (1.37,1.54)	< 0.001	2.06 (1.56,2.72)	< 0.001	1.72 (1.51,1.96)	< 0.001	1.76 (1.56,2.00)	< 0.001	1.71 (1.30,2.24)	< 0.001
Caucasian	10	14,688	14,548	1.45 (1.37,1.54)	< 0.001	2.06 (1.56,2.72)	< 0.001	1.72 (1.50,1.96)	< 0.001	1.76 (1.55,2.00)	< 0.001	1.71 (1.30,2.24)	< 0.001
East Asian	3	1544	1674	1.75 (0.49,6.29)	0.388	–	–	2.06 (0.38,11.3)	0.404	2.06 (0.38,11.3)	0.404	–	–
C3 rs2230205 (Associated allele vs. Reference allele: A vs. G)													
Overall	5	3302	2732	0.99 (0.89,1.11)	0.903	1.04 (0.77,1.42)	0.780	1.00 (0.80,1.23)	0.967	1.00 (0.81,1.22)	0.992	1.06 (0.81,1.37)	0.687
Caucasian	1	880	598	0.90 (0.66,1.23)	0.507	0.45 (0.12,1.60)	0.215	0.98 (0.69,1.39)	0.902	0.93 (0.66,1.32)	0.699	0.45 (0.13,1.60)	0.217
East Asian	4	2422	2134	1.01 (0.89,1.14)	0.903	1.10 (0.80,1.51)	0.546	1.01 (0.77,1.32)	0.967	1.04 (0.80,1.33)	0.787	1.10 (0.84,1.43)	0.497
C3 rs2250656 (Associated allele vs. Reference allele: G vs. A)													
Overall	5	3278	2632	0.90 (0.75,1.08)	0.257	0.76 (0.49,1.16)	0.207	0.78 (0.65,0.95)	0.014	0.78 (0.65,0.94)	0.010	0.83 (0.55,1.27)	0.391
Caucasian	1	874	512	0.82 (0.64,1.05)	0.117	0.77 (0.42,1.42)	0.407	0.76 (0.55,1.05)	0.097	0.76 (0.56,1.04)	0.085	0.87 (0.48,1.57)	0.642
East Asian	4	2404	2120	0.92 (0.73,1.16)	0.486	0.74 (0.41,1.37)	0.340	0.80 (0.63,1.02)	0.068	0.79 (0.63,1.00)	0.052	0.80 (0.44,1.45)	0.456

### Association between SNP rs2230199 of *C3* gene and advanced AMD

**Fig. 2:**
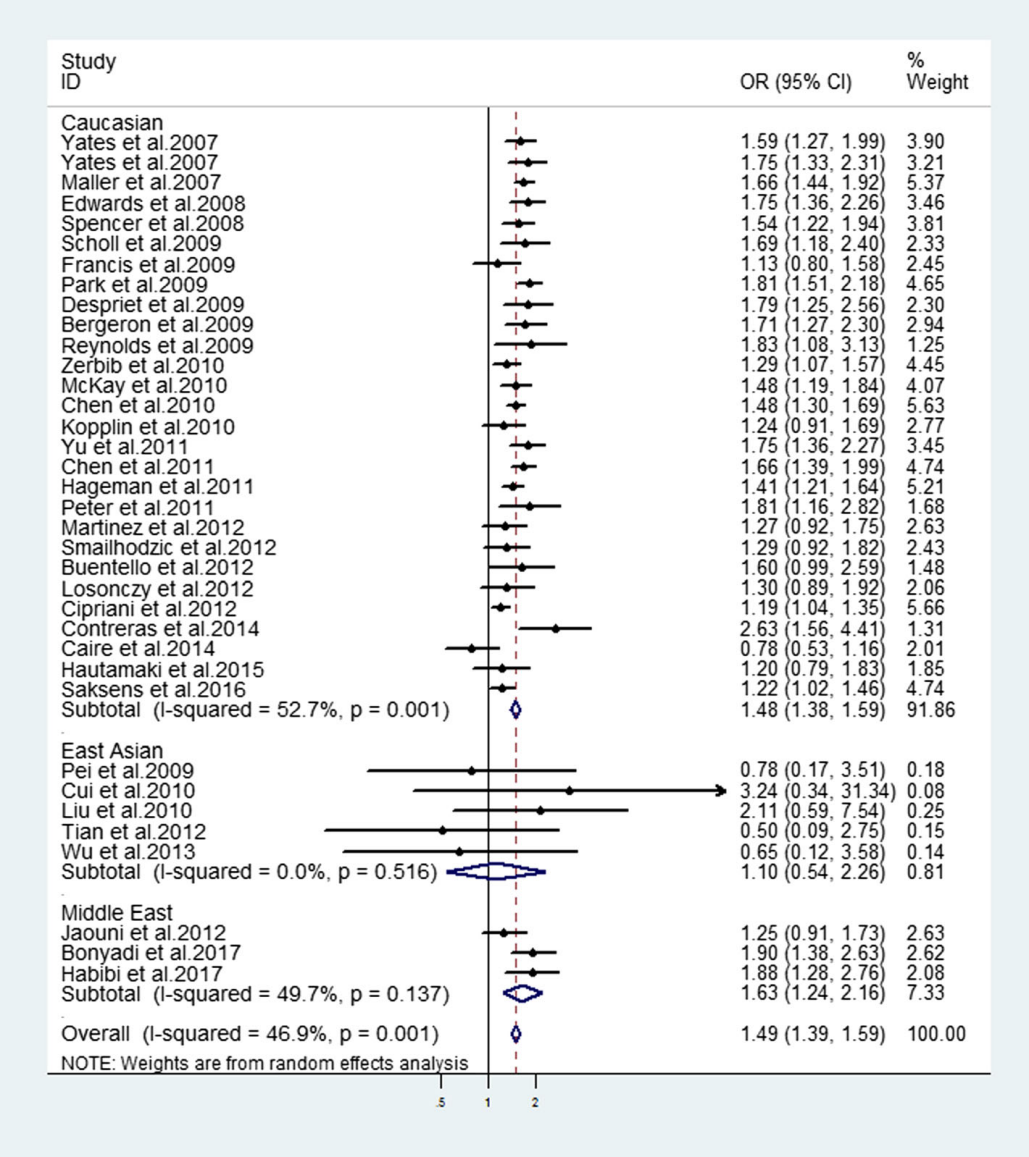
Evaluation of the association between *C3* genetic polymorphism (rs2230199) with advanced AMD

As shown in Table 3, there was a significant association between the rs2230199 SNP and advanced AMD susceptibility in the overall populations (allelic model: OR = 1.49, 95% CI = 1.39–1.59, *P* < 0.001; homozygote model: OR = 2.33, 95% CI = 1.98–2.74, P < 0.001; heterozygote model: OR = 1.53, 95% CI = 1.41–1.64, P < 0.001; dominant model: OR = 1.62, 95% CI = 1.51–1.74, *P* < 0.001; recessive model: OR = 1.99, 95% CI = 1.70–2.34, *P* < 0.001). Moreover, the subgroup analysis straitified by ethnicity indicated that rs2230199 conferred obvious susceptibility to advanced AMD in the group of Caucasian in allelic (OR = 1.48, 95% CI = 1.38–1.59, *P* < 0.001) (Fig. 2), homozygote (OR = 2.20, 95% CI = 1.87–2.60, *P* < 0.001), heterozygote (OR = 1.55, 95% CI = 1.43–1.67, P < 0.001), dominant (OR = 1.63, 95% CI = 1.51–1.75, P < 0.001), recessive (OR = 1.88, 95% CI = 1.59–2.21, P < 0.001) models (Table 3). Besides, the allelic comparison yielded a positive correlation in Middle East group (OR = 1.62, 95% CI = 1.33–1.97, *P* < 0.001). However, this relationship was not significant in East Asian group for any genetic models (Table 3).

### Association between SNP rs1047286 of *C3* gene and advanced AMD

**Fig. 3:**
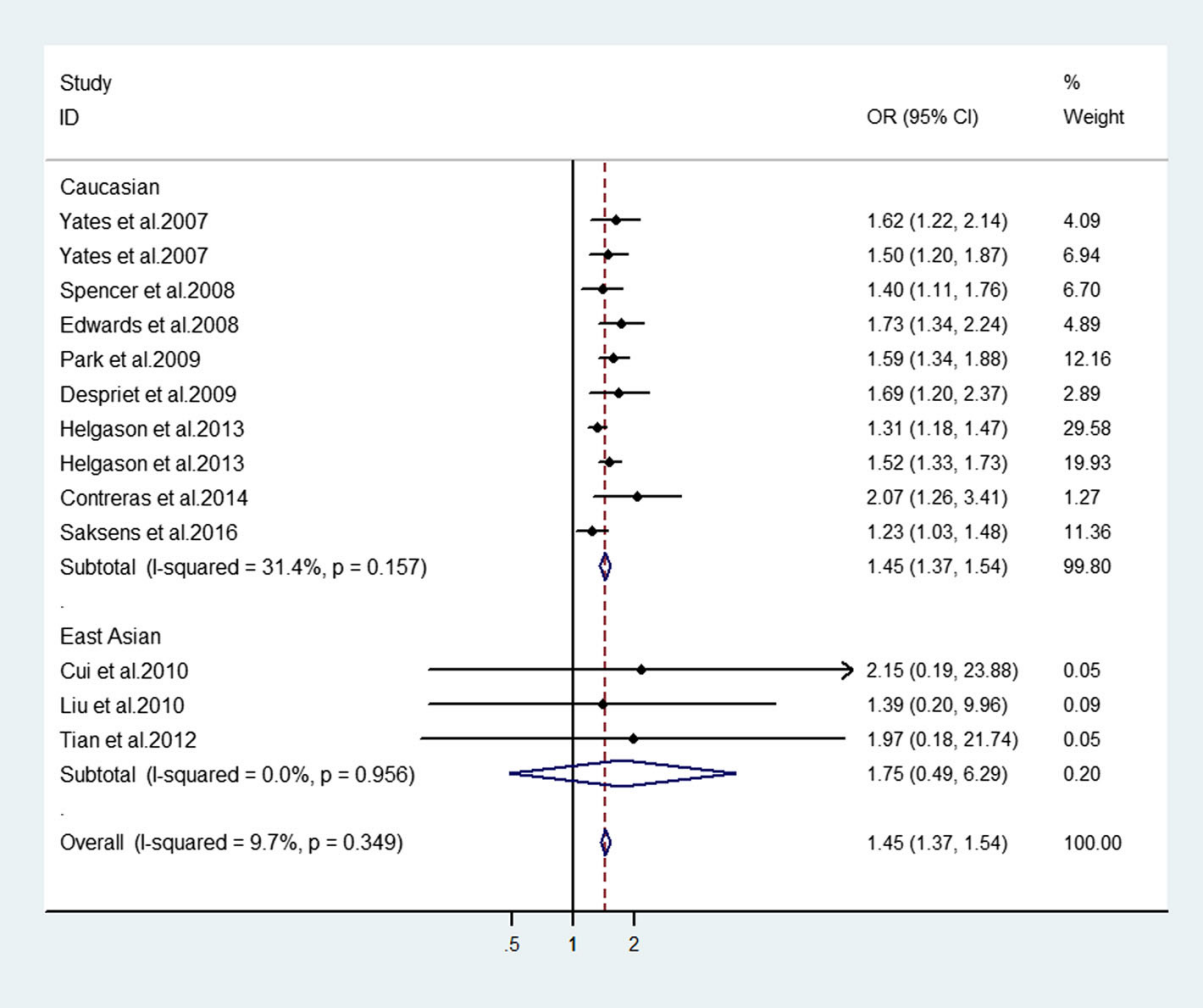
Assessment of the association between *C3* genetic polymorphism (rs1047286) with advanced AMD

Significant association between this SNP and advanced AMD was confirmed in the overall populations (allelic model: OR = 1.45, 95% CI = 1.37–1.54, *P* < 0.001; homozygote model: OR = 2.06, 95% CI = 1.56–2.72, *P* < 0.001; heterozygote model: OR = 1.72, 95% CI = 1.51–1.96, *P* < 0.001; dominant model: OR = 1.76, 95% CI = 1.56–2.00, *P* < 0.001; recessive model: OR = 1.71, 95% CI = 1.30–2.24, *P* < 0.001). In subgroup analysis stratified by ethnicity, our meta-analysis indicated significant correlation of rs1047286 with advanced AMD in the five genetic models (allelic model: OR = 1.45, 95% CI = 1.37–1.54, *P* < 0.001 (Fig. 3); homozygote model: OR = 2.06, 95% CI = 1.56–2.72, P < 0.001; heterozygote model: OR = 1.72, 95% CI = 1.50–1.96, P < 0.001; dominant model: OR = 1.76, 95% CI = 1.55–2.00, P < 0.001; recessive model: OR = 1.71, 95% CI = 1.30–2.24, P < 0.001) (Table 3). This association could not be found in East Asian group for any genetic model (Table 3).

### Association between SNP rs2230205 of *C3* gene and advanced AMD

**Fig. 4:**
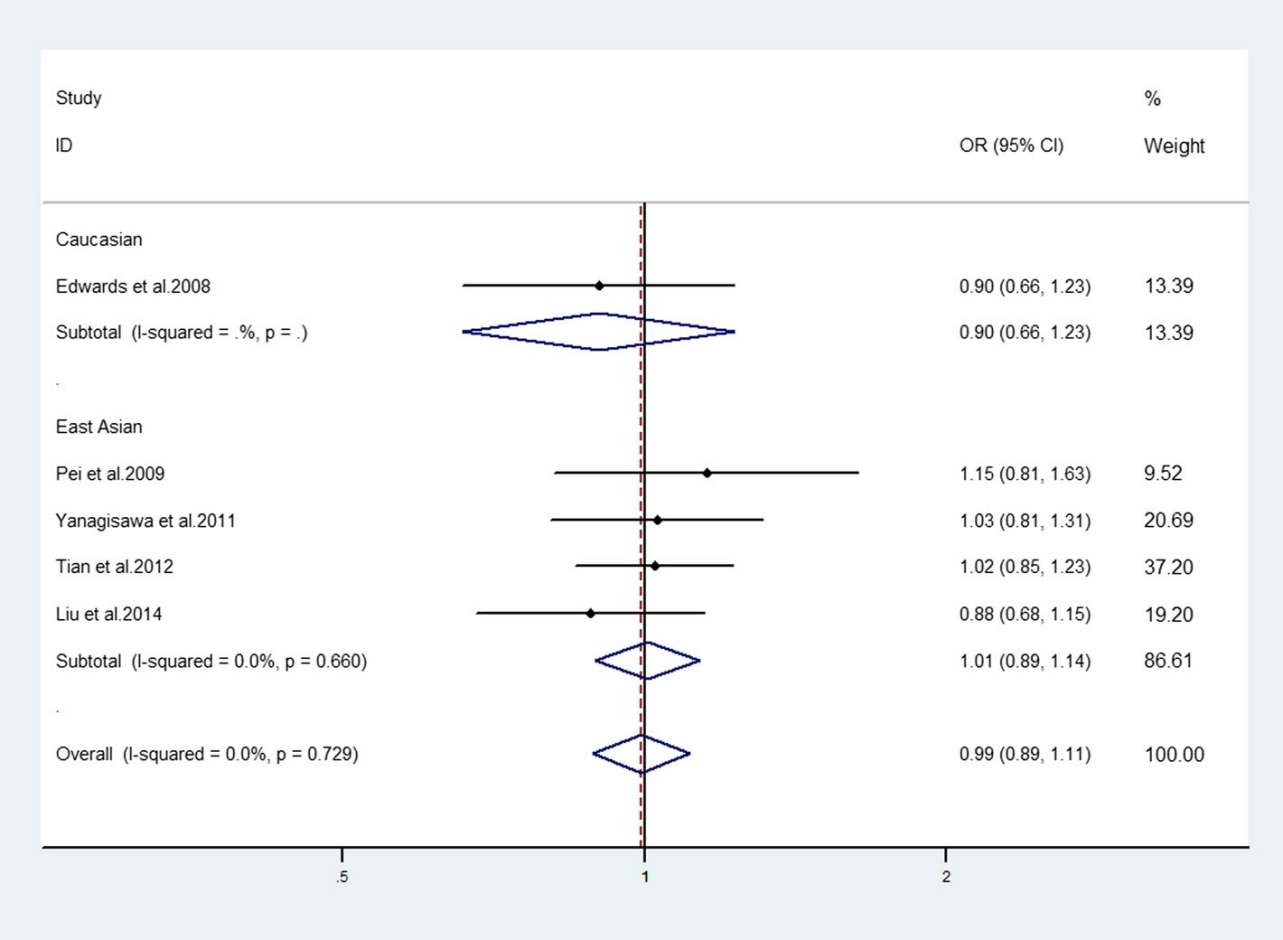
Estimation of the association between *C3* genetic polymorphism (rs2230205) with advanced AMD

No association between this SNP and advanced AMD was achieved in the overall populations (allelic model: OR = 0.99, 95% CI = 0.89–1.11, *P* = 0.903; homozygote model: OR = 1.04, 95% CI = 0.77–1.42, *P* = 0.780; heterozygote model: OR = 1.00, 95% CI = 0.80–1.23, *P* = 0.967; dominant model: OR = 1.00, 95% CI = 0.81–1.22, *P* = 0.992; recessive model: OR = 1.06, 95% CI = 0.81–1.37, *P* = 0.687). Subgroup analysis of Caucasian and East Asian group showed that there was a lack of relationship in any of the genetic models (Fig. 4, Table 3).

### Association between SNP rs2250656 of *C3* gene and advanced AMD

**Fig. 5:**
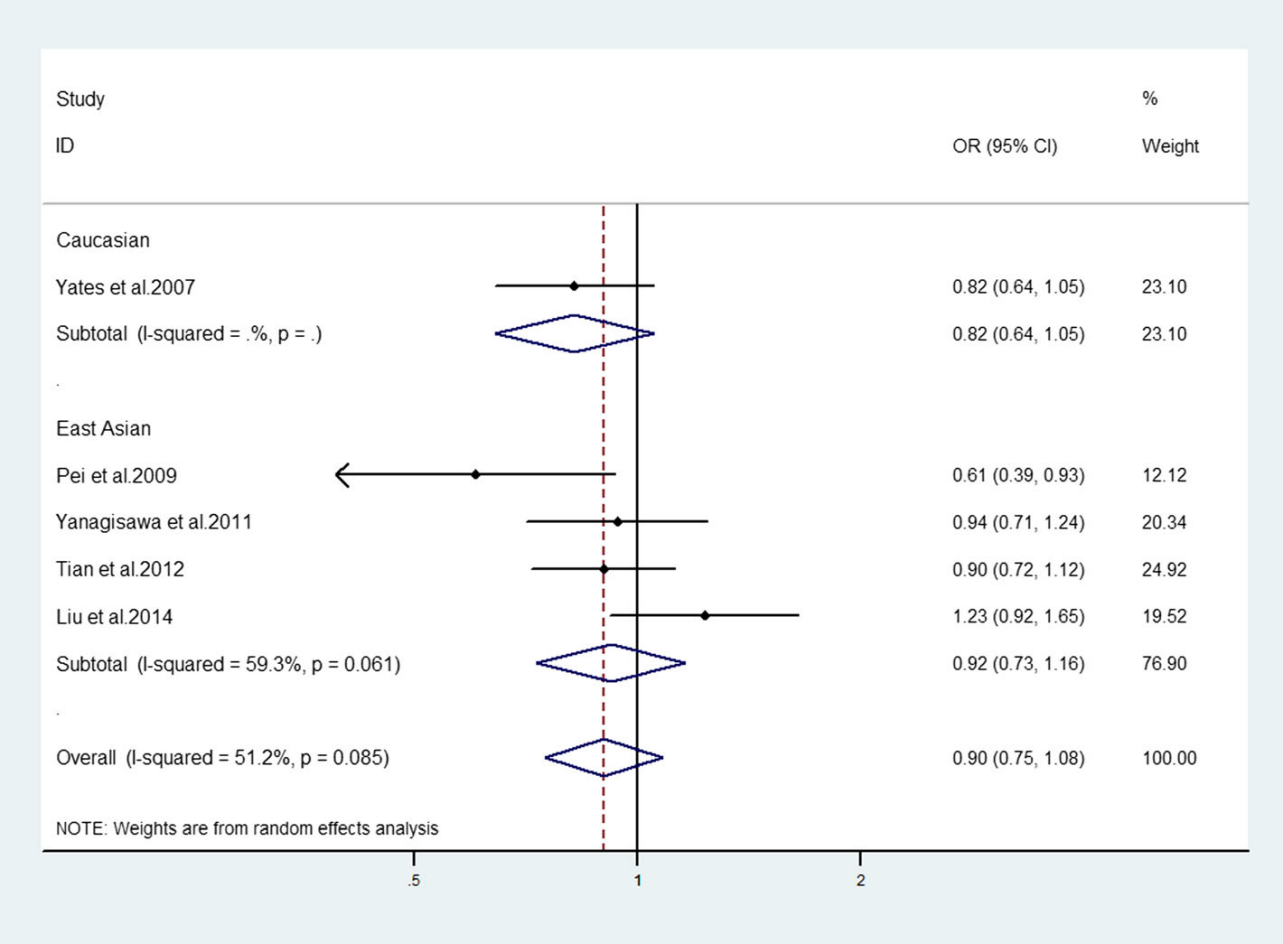
Evaluation of the association between *C3* genetic polymorphism (rs225065) with advanced AMD

The results of meta-analysis showed that there was not a positive association between this SNP and advanced AMD in the overall populations (allelic model: OR = 0.90, 95% CI = 0.75–1.08, *P* = 0.257; homozygote model: OR = 0.76, 95% CI = 0.49–1.16, *P* = 0.207; recessive model: OR = 0.83, 95% CI = 0.55–1.27, *P* = 0.391). But a weakly protective risk between this SNP and advanced AMD was observed in heterozygote model and dominant model (OR = 0.78, 95% CI = 0.65–0.95, *P* = 0.014; OR = 0.78, 95% CI = 0.65–0.94, *P* = 0.010, respectively). In the stratified analysis by ethnicity, there was no association in any of the genetic models. (Fig. 5, Table 3).

### Heterogeneity test and sensitivity analysis

**Fig. 6:**
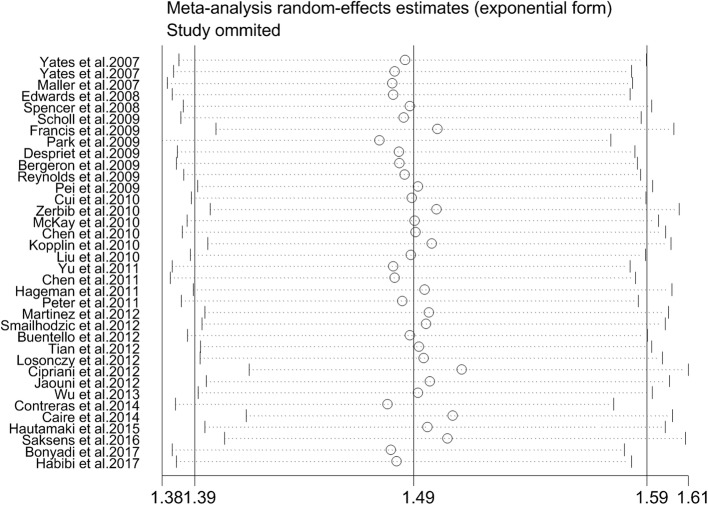
Evaluation of the sensitivity analysis between *C3* genetic polymorphism (rs2230199) with advanced AMD

Significant heterogeneity between these studies was observed among two SNPs (rs2230199 and rs2250656) (*P* < 0.1) (Figs. 2, 5). The results of our subgroup analysis confirmed that ethnicity was the primary sources of heterogeneity. Additionally, sensitivity analysis was conducted to evaluate the effect of individual study on the pooled ORs by sequentially omitting each study. The pooled ORs were not affected by removing any study (Fig. 6, the sensitivity analysis of rs2230199; others see Additional file 2: Figures S1-S3).

### Publication bias

Publication bias is a potential problem, thus Begg’s funnel plots and Egger’s regression tests were applied to investigate the publication bias for *C3* genetic polymorphism. Four symmetrical funnel plots suggested that both tests had no evidence of significant bias (data not shown). Furthermore, as emerged in Table 4, the pooled *P* values for both tests are more than 0.05.

**Table 4:** Bias between *C3* genetic polymorphism with advanced AMD in our Meta-Analysis

Polymorphism	Number of publication	Publication bias	
		Begg’s test	Egger’s test
rs2230199	36	0.653	0.790
rs1047286	13	0.428	0.124
rs2230205	5	1.000	0.905
rs2250656	5	1.000	0.594

## Discussion

AMD is a multifactorial disease, in which complement system mediated inflammation plays a pivotal role. Several pathways including the alternative complement component have been described to be implicated in the development of AMD [54]. As the central element of the complement cascade, *C3* has been a plausible candidate gene since its cleavage product C3a was confirmed in drusen. In our current meta-analysis, 20,673 patients and 20,025 controls from 38 articles were combined to detect the association of *C3* genetic polymorphisms with advanced AMD. We came to the conclusion that two nonsynonymous SNPs rs2230199 and rs1047286 were demonstrated an increased pathogenic effect on advanced AMD (rs2230199: allelic model: OR = 1.49, 95% CI = 1.39–1.59, *P* < 0.001; homozygote model: OR = 2.33, 95% CI = 1.98–2.74, P < 0.001; rs1047286: allelic model: OR = 1.45, 95% CI = 1.37–1.54, P < 0.001; homozygote model: OR = 2.06, 95% CI = 1.56–2.72, P < 0.001). Moreover, our meta-analysis discovered that SNP rs2250656 decreased the risk of advanced AMD susceptibility, which a protective association was acquired in heterozygote model and dominant model. Obviously, the results of SNP rs2250656 with advanced AMD needed to be validated with larger samples and studies in different ethnicity.

Being consistent with previous studies, the G allele of rs2230199 conferred susceptibility to advanced AMD in Caucasian group. In our meta-analysis, we first confirmed that the G allele of rs2230199 could be linked with AMD in Middle East but not East Asian region, though rather larger population needed to be validated in the future. Besides, our meta-analysis found a novel association between the T allele of rs1047286 and advanced AMD in Caucasian but not East Asian group.

The common polymorphisms rs2230199 and rs1047286 in the *C3* gene have been identified as genetic risk factors for advanced AMD in Caucasian populations. However, the allele frequencies of rs2230199 vary widely among different ethnicities. Frequencies of the risk G allele at rs2230199 were 25% to 31% in AMD cases and 19% to 21% in controls in Caucasians [29]. Besides, the frequencies of G allele was 14% to 25% in both cases and controls in Middle East region [20, 57]. While, the risk allele were absent in Japanese and rare (< 1%) in Chinese populations [53]. For rs1047286, frequencies of the risk T allele were 27% to 29% in AMD and 20% to 22% in controls in Caucasians. Cui et al. [29] also foud that rs1047286 was only 0.3% to 1% in both cases and controls and was not significantly associated with advanced AMD in Chinese population. The facts that rs2230199 and rs1047286 did not show tendency to risk of advanced AMD in East East but in Caucasian and Middle East could be explained by the lower minor allele frequencies (< 5%) of this two SNPs, suggesting that the susceptibility to advanced AMD by the variants of rs2230199 and rs1047286 did not transcend ethnic lines. In other words, this difference in the association between different ethnicities may result from other influence factors such as geography, the level of socioeconomic development or race.

The gene for *C3* is located on the short arm of chromosome 19 and consists of 41 exons, which forms 13 functional domains. *C3* is the most abundant complement component and significant *C3* messenger RNA is detected in the neural retina, choroid, RPE, and cultured RPE cells [58]. Cleavage of C3 into C3a and C3b is the central step in complement activation, which amplifies the complement response, resulting in the formation of lytic pores in the cell membrane. Janssen et al. [59] argued that cleaved native *C3* undergoes important structural rearrangements which causes conformational changes exposing binding sites for complement components and drusen including C3 and its activation products was confirmed in the finding that local inflammation and activation of the complement cascade can contribute to the pathogenesis of AMD. Notably, animal studies conducted by Bora et al. [60, 61] have indicated that *C3* deficiency in *C3*^−/−^ mice prevented the formation of choroidal neovascularization in advanced AMD (wet AMD), indicating that C3 is a pivotal element of this activation process.

In our meta-analysis, four SNPs including rs2230199, rs1047286, rs223205 and rs2250656 were analyzed in the pooled data. Among them, rs2230199 and rs1047286 are located in the first ring of macroglobulin domains, which conduct a prominent function for correct orientation of the thioester-containing domain. The amino acid changes induced by the genetic mutations may alter the configuration of the macroglobulin ring [62]. With evidence supporting a biologic functional effect through the formation of two electrophoretic allotypes in rs2230199 genetic site (*C3*F and *C3*S), the two alleles showed a differential capacity to bind monocyte- complement receptor. Helgason et al. [35] noted that the G allele in rs2230199 (*C3*F) was associated with the reduction of *C3* gene binding to CFH, which leads to an increase in complement activation. Additionally, rs2230199 variant may alter the net charge of the molecule and influence the position of the thioester-containing domain. Except for advanced AMD, the risk variant of rs2230199 has been previously considered as associated with other immune-mediated conditions, such as IgA nephropathy, systemic vasculitis.

In the current meta-analysis, rs1047286 variant showed significant association with advanced AMD in Caucasian populations. Despriet et al. [30] argued that rs2230199 and rs1047286 variants were in high linkage disequilibrium (LD) (D’ = 0.90, r^2^ = 0.80), which haplotype analyses suggested that the effect of the *C3* alleles was independent from the established genetic and environmental risk factors. Furthermore, our pooled analysis of neighboring SNPs of rs2230199 indicated that the allele frequency of the variant rs2230205 and rs2250656 was not significantly different between the advanced AMD cases and controls. Pei et al. [45] confirmed that the G allele of rs2250656 variant may be a protective factor for the development of AMD in East Asian. Given that the site of rs2250656 lies near the junction of intron 2 and exon 3, which contain short sequences and regulate the expression of gene and neighboring genes, it may contribute to the low risk for advanced AMD. Obviously, our pooled results were inconsistent with Pei’s report, owing to the relative small sample size and distinct environmental elements.

In a previous meta-analysis where a total of 15 independent studies with 5593 cases and 5181 controls were included, Zhang et al. [63] indicated that rs2230199 C > G SNP increased the risk of AMD development and the G allele was a risk factor for AMD in Caucasian but not Asians. Moreover, Yu et al. [64] have implemented a systemic meta-analysis and the overall results suggested a positive association between rs2230199, rs1047286 and AMD susceptibility. Additionally, Despriet et al. [30] have clarified these positive associations for only four available studies. In comparison to previous meta-analyses, our analysis mainly focused on the major form of AMD (advanced AMD) and was involved with a greater number of studies and larger sample size. These would make our pooled ORs more believable, stable, and accurate than before, especially in the association with advanced AMD. Moreover, our present meta-analysis encompassed an acceptable quality evaluation system, minimizing the potential bias.

Considerable efforts have been paid to discuss the potential relationship between *C3* genetic polymorphisms and advanced AMD, some limitations for our present meta-analysis need to be declared. First, heterogeneity among the ethnic groups was discovered when investigating the association of *C3* genetic variants with advanced AMD. However, based on the results of the sensitivity analysis, it is clear that the overall effect was not affected by heterogeneity. Additionally, there was no obvious publication bias detected in the contrast of *C3* gene with advanced AMD. Second, the number of patients and controls was relatively small in each included study; therefore, a great number of samples from different ethnic regions are required for further analysis. Third, the effects of common confounding factors, including sex, age, body mass index, smoking, and diet were not evaluated in the present study because of insufficient data. Fourth limitation is that only three ethnic backgrounds with relatively few studies were taken into consideration, thus further efforts to reduce the incidence of ethnic bias will be needed once raw data become available. Finally, the electronic databases from which we selected eligible studies were listed in English and Chinese; therefore, a language bias may be existed in our meta-analysis.

## Conclusion

The present meta-analysis provided a series of evidence-based pooled data for a significant association between rs2230199, rs1047286 and susceptibility to advanced AMD, especially in Caucasians. Additional well-designed work with a larger number of studies in which incorporate different ethnicities together with gene-gene and gene-environment is recommended to better confirm the functional role of the two nonsynonymous polymorphisms.

## Additional files

Additional file 1: The full details of databases searching terms. (DOC 38 kb)

Additional file 2: **Figures S1-S3.** The sensitivity analysis of C3 genetic polymorphisms (rs1047286,rs2230205,rs2250656). (DOC 106 kb)
