# Supplementary material for: Association between genetic variation of complement C3 and the susceptibility to advanced age-related macular degeneration: a meta-analysis
Source: BMC Ophthalmol. 2018 Oct 23;18:274. doi: 10.1186/s12886-018-0945-5 (PMC6199710; doi:10.1186/s12886-018-0945-5)
Supplement: Supplementary file 1 — The full details of databases searching terms. (DOC 38 kb) [file 12886_2018_945_MOESM1_ESM.doc]

## Additional file 1 Databases searching terms

## (updated to September 1^st^, 2018)

#### (1) Pubmed Database (n=236)

#1：(((((((((AMD) OR Age Related Maculopathies) OR Age Related Macular Degeneration) OR Age Related Macular Degenerations) OR Age Related Maculopathy) OR Macular Degeneration) OR Maculopathy) OR Maculopathies)) OR "Macular Degeneration"[Mesh]------34358

#2：(((((Complement component 3) OR Complement C3) OR Complement 3) OR C3)) OR "Complement C3"[Mesh]------48692

#3：((((((((((((((((genotype) OR Polymorphism) OR genentic) OR Polymorphisms) OR SNP) OR SNPs) OR single nucleotide polymorphism) OR genetic polymorphism) OR Mutation) OR Mutations) OR Variant) OR Variants) OR variation) OR variations) OR genetic Variant)) OR ((("Mutation"[Mesh]) OR "Polymorphism, Single Nucleotide"[Mesh]) OR "Polymorphism, Genetic"[Mesh])------2153362

#1 and #2 and #3: -----**236**

#### (2) EMBASE Database (n=328)

('AMD' OR 'Age Related Maculopathies' OR 'Age Related Macular Degeneration' OR 'Age Related Macular Degenerations' OR 'Age Related Maculopathy' OR 'Macular Degeneration' OR 'Maculopathy' OR 'Maculopathies') AND ('Complement 3' OR 'Complement C3' OR 'Complement component 3' OR 'C3') AND ('Genotype' OR 'genentic' OR 'Polymorphism' OR 'Polymorphisms' OR 'SNP' OR 'SNPs' OR 'single nucleotide polymorphism' OR 'genetic polymorphism' OR 'genetic Variant' OR 'Mutation' OR 'Mutations' OR 'Variant' OR 'Variants' OR 'variation' OR 'variations')------**328**

#### (3) Cochrane Library (n=36)

#1：MeSH descriptor: [Macular Degeneration] explode all trees-----**2068**

#2：MeSH descriptor: [Complement C3] explode all trees-----**281**

#3：MeSH descriptor: [Polymorphism, Genetic] explode all trees-----**2994**

#4：#1 and #2 and #3-----**36**

#### (4) Web of Science (n=564)

#1：(((((((((AMD) OR Age Related Maculopathies) OR Age Related Macular Degeneration) OR Age Related Macular Degenerations) OR Age Related Maculopathy) OR Macular Degeneration) OR Maculopathy) OR Maculopathies)) -----36129

#2：(((((Complement component 3) OR Complement C3) OR Complement 3) OR C3)) ------49346

#3：((((((((((((((((genotype) OR Polymorphism) OR genentic) OR Polymorphisms) OR SNP) OR SNPs) OR single nucleotide polymorphism) OR genetic polymorphism) OR Mutation) OR Mutations) OR Variant) OR Variants) OR variation) OR variations) OR genetic Variant)) ------2163524

#1 and #2 and #3: -----**564**
