# Supplementary material for: Association between genetic variation of complement C3 and the susceptibility to advanced age-related macular degeneration: a meta-analysis
Source: BMC Ophthalmol. 2018 Oct 23;18:274. doi: 10.1186/s12886-018-0945-5 (PMC6199710; doi:10.1186/s12886-018-0945-5)
Supplement: Supplementary file 2 — Figures S1-S3. The sensitivity analysis of C3 genetic polymorphisms (rs1047286,rs2230205,rs2250656). (DOC 106 kb) [file 12886_2018_945_MOESM2_ESM.doc]

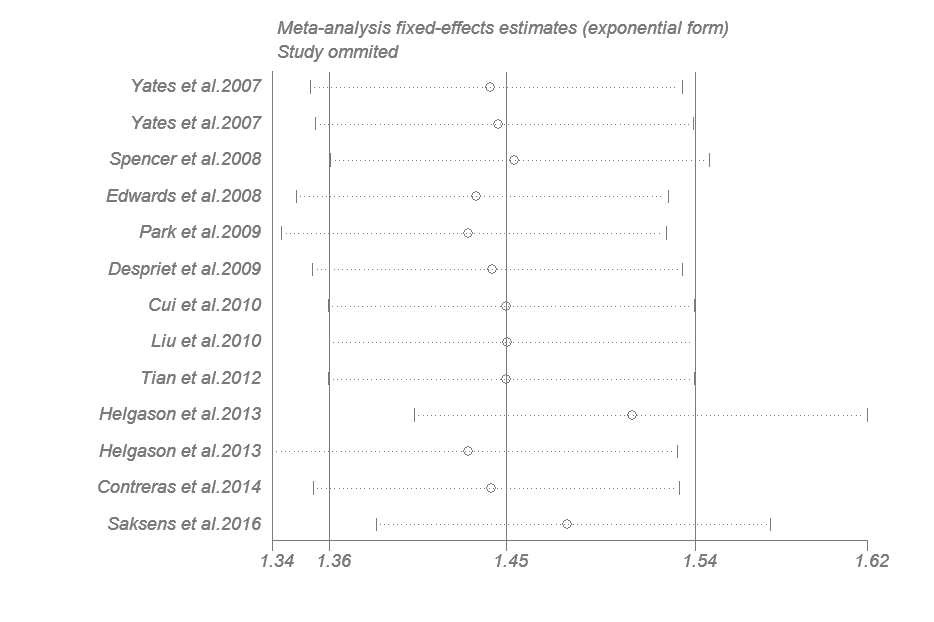


Supplement Figure 1| Evaluation of the sensitivity analysis between *C3* genetic polymorphism (rs1047286) with advanced AMD.


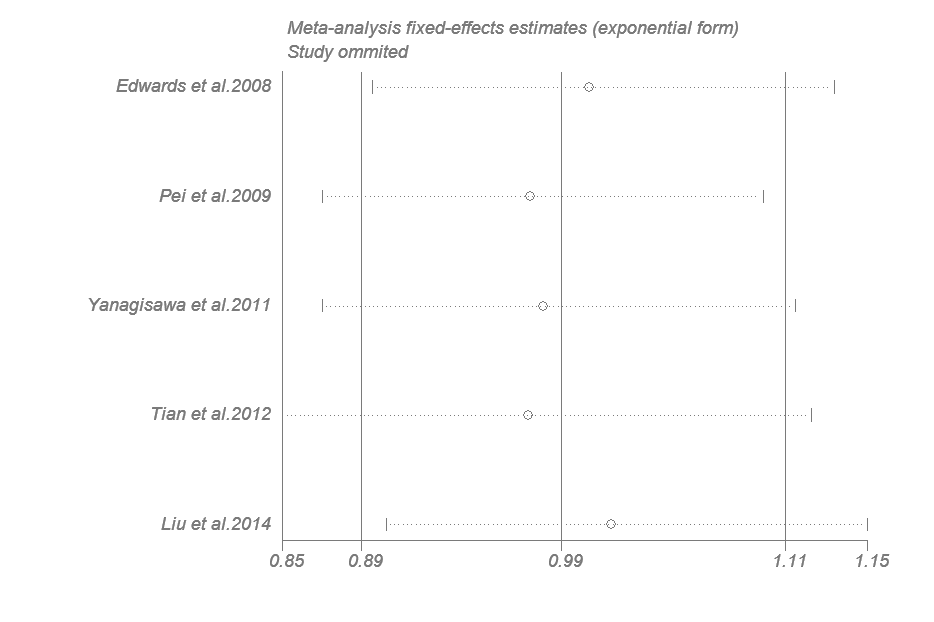


Supplement Figure 2| Assessment of the sensitivity analysis between *C3* genetic polymorphism (rs2230205) with advanced AMD.


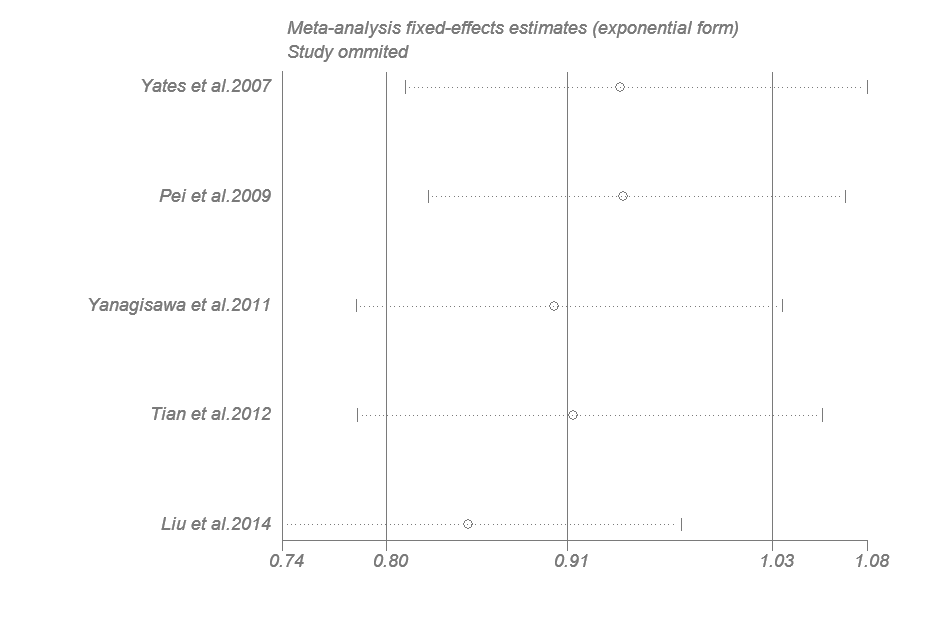


Supplement Figure 3| Estimation of the sensitivity analysis between *C3* genetic polymorphism (rs2250656) with advanced AMD.
